# Applying the Theory of Planned Behavior to healthy eating behaviors in urban Native American youth

**DOI:** 10.1186/1479-5868-3-11

**Published:** 2006-05-30

**Authors:** Stefanie A Fila, Chery Smith

**Affiliations:** 1 Albuquerque School System, 1304 Sierra Larga NE, Albuquerque, NM 87112, USA; 2 University of Minnesota, Department of Food Science and Nutrition, 225 FScN, 1334 Eckles Ave, St. Paul, MN 55108–6099, USA

## Abstract

**Background:**

To investigate the efficacy of the Theory of Planned Behavior (TPB) to predict healthy eating behavior in a group of urban Native American youth.

**Methods:**

Native American boys and girls (n = 139), ages 9–18 years old, were given a self-administered survey to assess eating behavior using the TBP constructs (intention, attitude, subjective norm, barriers, self-efficacy, and perceived behavioral control). Youth were also measured for height and weight and body mass index (BMI) was calculated. Bivariate correlations and stepwise regression analyses of TBP model were performed with SPSS software.

**Results:**

No association was found between intention and healthy eating behavior. However, independently healthy eating behavior was correlated with barriers (0.46), attitude (0.44), perceived behavioral control (0.35), and subjective norm (0.34). The most predictive barriers to eating healthy included the availability and taste of foods. Boys' eating behavior was most predicted by subjective norm, while girls' eating behavior was most predicted by barriers.

**Conclusion:**

Lack of association between intention and healthy eating behavior suggests that factors other than intentions may drive healthy eating behaviors in urban Native American youth. Results indicate that programs promoting healthy eating to youth might focus on collaborating with families to make healthy foods more appealing to youth.

## Background

Several studies suggest that Native American youth have a higher prevalence of obesity than the general United States population [1-7]. The problem is not restricted to just Native American youth, increases in pediatric obesity have been noted globally [8]. Additionally, research suggests that obesity may persist into adulthood and increase the risk of chronic diseases including heart disease, increased blood pressure, and type 2 diabetes, thus making it a major public health concern [9,10]. Because of the known association between dietary intake and obesity [11,12], promoting healthy eating behaviors in youth could help decrease the elevated prevalence of obesity. While little is know about the dietary habits of urban Native American youth, research by Ballew et al. [13] found that 12–19 year-old rural youth participating in the Navajo Health and Nutrition Survey consumed fruits, vegetables, and dairy products less than once per day and mean intakes of several vitamins and minerals were below the sex-and-age specific recommended dietary allowances. Because preliminary research indicates poor dietary intake and a high prevalence of obesity among Native American youth, it seems prudent to examine whether youth have an interest in or intend to eat healthfully and to identify the factors that influence those intentions so that appropriate nutrition intervention programs could be developed to assist youth in changing their dietary behavior. Therefore, the purpose of this project was to learn more about why urban Native American youth eat the way that they do. Specifically, we were interested in identifying attitudes that promote, or create barriers, to healthful eating; identifying who or what promotes healthful dietary behavior; and examining to what extent the youth perceive control over their dietary behavior. We utilized the Theory of Planned Behavior (TPB) as the theoretical framework to accomplish this.

The TPB is often used to study health related decision making behavior in youth [14-19]. The TPB is an extension of the Theory of Reasoned Action (TRA) [20], but incorporates a third construct known as perceived behavioral control (PBC). The TPB model suggests that intention is directly driven by three major constructs attitude, subjective norm, and PBC and the stronger the intention, the more likely an individual will perform the behavior [21]. Attitude is known as the degree to which an individual has a favorable or unfavorable evaluation of the behavior, subjective norm measures the importance others hold about performing or not performing a behavior and one's willingness to comply to those referents, and PBC describes the perceived ease or difficulty an individual has for performing a behavior. In addition, PBC is thought to directly affect behavior by accounting for factors outside an individual's control and especially for behaviors not under volitional control [22]. Like many health behaviors, healthy eating is not under complete volitional control, as a result, perceived behavioral control becomes a more important determinant of behavior [23].

Self-efficacy is another term that has sometimes been used to define PBC. Self-efficacy is a component of Bandura's [24] Social Cognitive Theory and is defined as an individual's perceived ability to perform a behavior. Ajzen [23] considers the PBC construct of TPB identical to self-efficacy. However, researchers have not yet come to agreement about this. Self-efficacy has been shown to be an independent contributor to eating behavior [25] and several studies support a distinction between self-efficacy and PBC when applying the TPB to health behaviors [26-28]. Armitage and Conner [27] applied the TPB to eating a low-fat diet in a group of undergraduate students. They differentiated self-efficacy, the individual's internal motivation to eat a low fat diet, from PBC, the extent to which an individual has control over external factors related to eating a low fat diet [27]. In another study, Armitage and Conner [27] found that when self-efficacy was added to the TPB model, it was not only an important contributor but it was often the most important predictor of both behavior and intention. Giles et al. [29] suggested that the predictive utility of the TPB may be enhanced by replacing PBC with self-efficacy. Alternatively, some research indicates that self-efficacy is not a useful addition to the theory. For example, Terry and O'Leary [28] suggest that self-efficacy may not be an important predictor of behavior. Based on previous research findings, this current study examined self-efficacy and PBC as separate constructs that could indirectly, through intention, or directly influence healthy eating behavior in urban Native American youth.

Previous research has also investigated adolescents' knowledge and perceptions of healthy eating and found that while adolescents are informed about healthy eating practices and recommendations, they find it difficult to follow recommendations and often ate unhealthy foods because they perceived too many barriers to eat healthfully [30,31]. According to the Health Belief Model, perceived barriers are an individual's opinion of the tangible costs of an action or behavior [32]. In addition to the Health Belief Model, perceived barriers were also used as a construct to determine how well the TBP predicted fruit and vegetable consumption in adolescents [15]. Since perceived barriers appear to be a determinant of healthy eating behavior, and may also indirectly affect intention, this study also included barriers as a construct in the TBP model.

**Figure 1:**
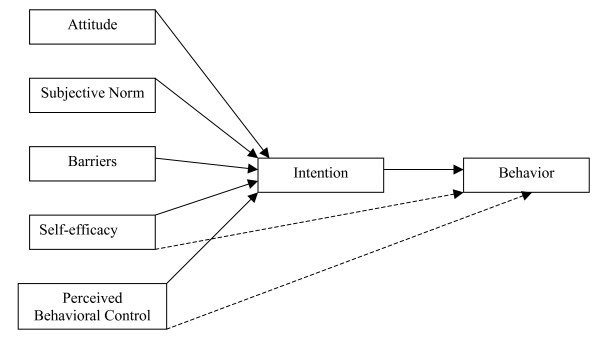
Study model of healthy eating based on the TPB.

This current study used an expanded TBP model which incorporates the original constructs of attitude, subjective norm, and PBC, as well as two additional constructs, barriers and self-efficacy, to investigate healthy eating behaviors in urban Native American youth (Figure 1). Because of the high prevalence of obesity among Native American youth, and the association between diet and weight gain, results from this study are important for the development of intervention strategies that promote healthy eating behaviors in urban Native American youth who are overweight or at risk for becoming overweight.

## Methods

### Participants

Participants were 139 urban Native American youth, predominately from Ojibwe and Lakota tribes, attending an after school program in Minneapolis, Minnesota. The goal of the program was to promote cultural identity, academic success, physical well being, and chemical dependency awareness to Native American youth ages 5 to 18 years old. For this study, surveys were self-administered only to youth 9 to 18 years. This sample included 58 boys (mean age: 12.5 + 2.6 yrs) and 81 girls (mean age: 12.4 + 2.4). Parental consent was given for all activities at the time of program enrollment, and youth were asked to complete the survey voluntarily. Youth who completed the survey and anthropometric measurements received a modest monetary incentive for their participation. Of the approximately 150 youth (ages 9 to 18 yrs) attending the program at the time of this project, 93% volunteered to participate. The project was approved by the Institutional Review Board of Human Subjects at the University of Minnesota.

### Measures

The survey was developed according to procedures defined by Ajzen and Fishbein [20]. Formative assessment included a review of literature and six focus group discussions with 39 youth, ages 9 to 18 years, to determine common beliefs about eating healthy, advantages and barriers to eating healthy, and important people who may influence behavior. Focus groups were audio taped and transcribed verbatim and analyzed for common themes. The themes were used to develop a survey using the constructs of the TPB to investigate healthy eating behavior. These include the original constructs of attitudes, subjective norm, PBC, as well as two additional constructs, self-efficacy and barriers (Figure 1). Self-efficacy was included to assess whether it was more predictive of intention and/or behavior than PBC. The construct 'barriers' was included in the model because the staff of the program were particularly interested in identifying factors that prevent youth from eating healthfully. The survey included 90 questions pertaining to eating behavior and measured all constructs. It was evaluated by an expert panel of nutritionists accomplished in the areas of survey development and behavioral theory for face validity and breadth of coverage and then pilot tested in a group of urban Native American youth (n = 32) attending an alternative high school for ease of comprehension and readability. The final version of the survey contained slight revisions in wording and question ordering based on recommendations from professionals and findings from the pilot study. Scales for the final survey were assessed using the Cronbach alpha coefficient, an index of inter-item homogeneity (internal consistency.) The reliability levels for the: attitude scale was 0.66, subjective norm scale was 0.66, PBC scale was 0.80, intention scale was 0.84, behavior scale was 0.72, barriers scale was 0.89, and self-efficacy scale was 0.85. These scores indicate a substantial (0.61 – 0.80) to almost perfect (0.81–1.0) range of reliability [32].

Survey questions were assessed using a five-point Likert response scale, youth had to choose one of the following responses "strongly agree", "agree", "unsure", "disagree", or "strongly disagree". For positively scaled questions responses were coded from 2 to -2 (strongly agree to strongly disagree) and for negatively scaled questions responses were coded from -2 to 2 (strongly agree to strongly disagree). Attitudes to eating healthy were measured by using the responses to eighteen questions about the importance of eating healthy foods, fruits, vegetables, regular pop, junk food, and fast food, and perceptions of eating healthy, being under or overweight, and diabetes (e.g. *"It is important to me to eat healthy foods everyday"*). Subjective norm was measured by using the responses to eight questions asking if parents, friends, elders, community programs, or television told youth to eat healthy everyday (e.g.*"My parents tell me it is important to eat healthy everyday"*). PBC measured external factors that may directly or indirectly affect healthy eating behavior. Responses to eight questions concerning youth's perceived control over eating healthy, eating junk food, drinking regular pop, eating fast food, eating in front of the TV, and getting diabetes, as well as having fruits and vegetables available (e.g. "*I have control over whether or not I eat healthy"*) were used to determine perceived behavior control. Intention to eat healthy was measured by using the responses to eight questions regarding youth's plans for the next week to eat healthy, eat vegetables, eat fruit, not eat junk food, not eat fast food, not drink regular pop, eat healthy foods in front of the TV, and eat healthy foods to keep a healthy weight everyday (e.g. "*For the next week I plan to eat healthy everyday"*). Eating behavior was measured by using the responses to questions assessing dietary intake of vegetables, fruits, soft drinks, and fast food consumption, along with eleven additional behavior questions using the Likert-scale. Eating behaviors questions included general questions such as, "I mostly eat healthy foods," "I eat healthy to keep me from getting diabetes," and "I eat junk food when I watch TV," but also used specific foods such as "fruits" and "vegetables" because youth defined foods, especially fruits and vegetables, as being healthy. Both healthy and junk foods were defined on the survey using terminology that the youth used during the focus group discussions. The assessment and behavioral question scales were were averaged, recoded, and the sum of the two were calculated to measure youth's behavior.

The two additional constructs, barriers and self-efficacy, were included in the expanded model. Barriers to eating healthy were measured by using the responses to fourteen questions concerning youth's perceptions about the taste of fruits, vegetables, regular pop, and junk food; the ease of eating healthy away from home, with friends, with family, in front of the TV and to keep a healthy weight; and availability of healthy foods (e.g. *"It is hard for me to eat healthy foods at fast food restaurants"*). Self-efficacy measured internal factors that may directly or indirectly affect healthy eating behavior. Responses to fifteen questions determining youth's ability to eat healthy foods and choose specific healthy foods (fruit, vegetables, chocolate milk, juice, white milk, low-fat milk, salads) over specific unhealthy foods (junk food, chips/cheetos, regular pop, chocolate milk, whole milk, hamburgers) (e.g. *"I can eat healthy foods everyday"*) were used to assess self-efficacy to eat healthy.

### Survey administration

Youth agreeing to participate in the study were seated in a quiet area and given a survey and writing instrument. Researchers explained to each youth that the purpose of the survey was to find out about how they feel about healthy eating and stressed that there were no right or wrong answers. Definitions for healthy eating, junk food, and fast food were provided on each survey and read aloud to each youth. These terms were identified and defined by youth attending the focus group discussions. Healthy eating was defined as eating different types of food from all food groups like bread, grains, cereals, fruit, vegetables, milk, and meat while limiting sugary and fatty foods; junk food was defined as foods such as pop, chips, candy, donuts, cakes, cookies, and sweets; and fast food was defined as burgers, fries, shakes and foods from McDonald's, Burger King, Taco Bell, KFC etc. Youth were asked to carefully read each question and mark only one response. For youth who had problems answering questions or understanding questions, several researchers were available to aid with completion. The time taken to complete each survey averaged 20 minutes with a range between 10 and 30 minutes. All surveys were rechecked to identify omitted or multiple response questions and youth were immediately instructed to finish or clarify unfinished or unclear responses. Four surveys were removed from analysis because of missing data.

### Anthropometric measurements

After completing the survey each respondent was measured in light clothing with their shoes removed. Height was measured to the nearest one-tenth centimeter with a GPM anthropometer (Switzerland). Weight was measured to the nearest one-tenth kilogram with a high-quality electronic scale (Seca, France). Body mass index (BMI) was calculated by dividing weight in kilograms by height in meters squared and the BMI for each youth was plotted on age-sex-specific growth charts developed by the Centers for Disease Control and Prevention [33]. Youth were classified according to their BMI percentile as either underweight (<5^th ^percentile), normal weight (≥ 5^th ^to <85^th ^percentile), at risk for overweight (≥ 85^th ^to <95^th ^percentile), or overweight (≥ 95^th ^percentile) [34].

### Data analysis

Data were analyzed using the Statistical Package for Social Sciences for Windows (SSPS, v. 11, Chicago, IL, 1999). Descriptive statistics were used to determine means and standard deviations of all constructs as well as age, gender, and BMI. Mean values for the constructs for boys and girls were compared using independent t-tests to identify any gender or age differences. No differences were found based on age.

Because gender differences were observed all analyses were conducted by gender. Pearson correlations were conducted to examine associations between the psychosocial variables (constructs) for both the original TPB model, then for the expanded TPB model. Because the PBC construct was only weakly associated to girl's behavior and not associated to girl's intention or boy's behavior or intention, the expanded version of TPB model was used for further analyses. In order to examine constructs (attitude, subjective norm, PBC, self-efficacy, and barriers) most predictive of intention and behavior, stepwise regression analyses were performed respectively. Additionally, further stepwise regression analyses were conducted with the construct that was most predictive of behavioral change for boys (subjective norms) and girls (barriers). Both of these issues will need to be addressed in future interventions if behavioral change is to be achieved. The level of significance was set at *p *< 0.05 for all statistical tests.

## Results

The mean age and grade of boys and girls was not significantly different, 12.4 years old and 7^th ^grade respectively. According to CDC BMI percentiles 38% of girls and 38% of boys were classified as normal weight, 20% of girls and 26% of boys as at risk for overweight, and 42% girls and 36% of boys as overweight.

### Boys

No association was found between intention and healthy eating behavior. Furthermore, the grade, age, and BMI were not associated with intention or behavior. However, the constructs of attitude, subjective norm, PBC, and barriers were all associated with behavior, and the constructs of attitude, subjective norm, and self-efficacy were all associated with intention (see Table 1). The mean value of barriers for boys (-0.006) was significantly higher than the mean value of barriers for girls (-0.032) (Table 1).

**Table 1:** Correlations between TBP model constructs. Means and standard deviations (SD) for each construct for the total sample and by gender.

	1	2	3	4	5	6	7
Total sample (n = 139)							

1. Behavior	1						
2. Intention	0.05	1					
3. Attitude	0.44**	0.45**	1				
4. Subjective norm	0.34**	0.39**	0.48**	1			
							
5. Barriers	0.46**	-0.14	0.40**	0.15	1		
6. Self-efficacy	-0.12	0.56**	0.38**	0.37**	-0.31**	1	
7. Perceived behavioral control	0.35**	-0.01	0.35**	0.23**	0.57**	-0.04	1
							
Mean	0.048	0.062	0.023	0.067	-0.021	0.034	0.003
SD	0.034	0.093	0.023	0.079	0.054	0.043	0.097
							
Boys (n = 58)							
1. Behavior	1						
2. Intention	0.15	1					
3. Attitude	0.45**	0.60**	1				
4. Subjective norm	0.46**	0.38**	0.58**	1			
5. Barriers	0.37**	0.13	0.47**	0.37**	1		
6. Self-efficacy	0.2	0.68**	0.65**	0.38**	0	1	
7. Perceived behavioral control	0.43**	0.22	0.46**	0.33*	0.59**	0.18	1
							
Mean	0.05	0.032†	0.023	0.066	-0.006†	0.030†	0.019
SD	0.032	0.09	0.028	0.086	0.056	0.045	0.096
							
Girls (n = 81)							
1. Behavior	1						
2. Intention	0.01	1					
3. Attitude	0.46**	0.34**	1				
4. Subjective norm	0.25*	0.41**	0.37**	1			
5. Barriers	0.52**	-0.26*	0.35**	-0.5	1		
6. Self-efficacy	-0.34**	0.41**	0.08	0.38**	-0.54**	1	
7. Perceived behavioral control	0.28*	-0.11	0.25*	0.16	0.53**	-0.16	1
							
Mean	0.046	0.084†	0.023	0.067	-0.032†	0.046†	-0.009
SD	0.036	0.089	0.02	0.075	0.05	0.039	0.096

* *p *< 0.05 ** *p *< 0.01 † Mean value significantly different than mean value of boys/girls at *p *< 0.05.

Stepwise regression of behavior as the dependent variable and constructs (attitudes, subjective norms, PBC, barriers, and self-efficacy) as independent variables, showed that subjective norm (R^2 ^= 0.21) and perceived behavioral control (R^2 ^= 0.30) predicted 30% of the model (Table 2). Whereas when intention was regressed with the constructs as independent variables, self-efficacy accounted for 46% of the variance and attitude added another 4%. Furthermore, because subjective norm was most predictive of healthy eating behaviors we ran it as a dependent variable with all survey questions measuring subjective norm as independent variables to see which of the social norms were most predictive of that construct. Three questions explained 86% of the variance in behavior (Table 2). The three questions included *"My family tells me I should eat healthy everyday to help keep me at a healthy weight" *(R^2 ^= 0.57), *"I get hungry for foods I see on TV" *(R^2 ^= 0.74), and *"The after school program I attend says it is important to eat healthy everyday" *(R^2 ^= 0.86).

**Table 2:** Stepwise regression analyses of healthy eating behavior.

**Variable**	**B**	**Standard error**	**R Square**	**P Value**
**Boys and Girls**				
Dependent variable: Behavior^a^				
Barriers	0.121	0.058	0.207	0.038
Attitude	0.519	0.142	0.286	0.000
Self-efficacy	-0.229	0.074	0.315	0.002
Subjective norm	0.105	0.035	0.357	0.004
Dependent variable: Barriers^b^				
Healthy foods not around	0.006	0.000	0.614	0.000
Healthy foods don't fill you up	0.005	0.000	0.762	0.000
Junk food taste better than healthy food	0.005	0.000	0.830	0.000
Friends make it hard to eat healthy	0.006	0.000	0.882	0.000
Fruits don't taste good	0.005	0.000	0.913	0.000
**Boys**				
Dependent variable: Behavior^a^				
Subjective norm	0.133	0.045	0.212	0.004
Perceived behavioral control	0.104	0.040	0.299	0.011
Dependent variable: Subjective norm^c^				
Family says eat healthy to keep weight	3.125E-02	0.000	0.565	0.000
I get hungry for foods on TV	1.563E-02	0.000	0.735	0.000
After school program says eat healthy	1.562E-02	0.000	0.862	0.000
Friends say eat healthy	1.563E-02	0.000	0.927	0.000
**Girls**				
Dependent variable: Behavior^a^				
Barriers	0.173	0.082	0.267	0.037
Attitude	0.538	0.187	0.354	0.005
Self-efficacy	-0.310	0.104	0.388	0.004
Subjective norm	0.134	0.047	0.446	0.006
Dependent variable: Barriers^b^				
Healthy foods are not around	5.102E-03	0.000	0.573	0.000
Parent's don't buy healthy foods	5.102E-03	0.000	0.738	0.000
Junk food taste better than healthy food	5.102E-03	0.000	0.819	0.000
Healthy foods don't fill you up	5.102E-03	0.000	0.868	0.000
Hard to eat healthy away from home	5.102E-03	0.000	0.907	0.000

^a ^Candidate variables: intention, attitude, subjective norm, barriers, self-efficacy, perceived behavioral control.

^b ^Candidate variables: fruits don't taste good, vegetables don't taste good, regular pop tastes better than diet, healthy foods don't fill you up, hard to eat healthy at fast food restaurants, hard to eat healthy away from home, hard to eat healthy because junk food tastes better, I am not use to eating healthy, healthy foods are not around, parent's don't buy healthy food, family makes it hard to eat healthy, friends make it hard to eat healthy, TV makes it hard to eat healthy, hard to eat healthy to keep at a healthy weight.

^c ^Parents say eat healthy, after school program says eat healthy, friends say eat healthy, Elders say eat healthy, no one tells me not to eat junk food, I get hungry for foods on TV, family says eat healthy to keep weight, family says eat healthy to prevent diabetes.

### Girls

No association was found between intention and behavior, therefore factors directly affecting behavior and intention were investigated. Healthy eating behavior was positively correlated with attitude, subjective norm, perceived behavior control, and barriers, while self-efficacy had a negative correlation (Table 1). While the constructs of attitude, subjective norm, and self-efficacy were all positively associated with intention, and barriers was negatively associated with it (see Table 1). The mean value of healthy eating intention (0.084) and self-efficacy (0.046) for girls was significantly higher than the mean value of intention (0.032) and self-efficacy (0.030) for boys (Table 1).

Stepwise regression of behavior as the dependent variable and constructs (attitudes, subjective norms, PBC, barriers, and self-efficacy) as independent variables, showed that barriers (R^2 ^= 0.27), attitude (R^2 ^= 0.35), self-efficacy (R^2 ^= 0.39), and subjective norm (R^2 ^= 0.45) predicted 45% of the model (Table 2). Whereas when intention was regressed with the constructs as independent variables, subjective norm (R^2 ^= 0.18), attitude (R^2 ^= 0.30), and barriers (R^2 ^= 0.35) predicted 35% of the variance (Table 2). Furthermore, because barriers was most predictive of healthy eating behaviors, we ran it as a dependent variable with all survey questions measuring subjective norm as independent variables to see which of the barriers were most predictive of that construct. Four questions were identified as explaining 82% of the variance in barriers to eat healthy (Table 2). The four questions included *"I don't eat healthy because healthy foods are not around" *(R^2 ^= 0.57), *"My parent's don't buy healthy food" *(R^2 ^= 0.74), *"It is hard for me to eat healthy foods because junk foods taste better" *(R^2 ^= 0.82), and *"Healthy foods don't fill you up" *(R^2 ^= 0.87).

## Discussion

In this investigation of the healthy eating behaviors in urban Native American youth, TPB was found to be predictive of factors affecting healthy eating intention and behavior independently, but found no direct association between intention to eat healthfully and eating behavior. Furthermore, gender, but not age, grade, or BMI, was found to be a significant factor in youths' responses. Among boys, intention and behavior were predicted by different constructs, with self-efficacy accounting for 46% of the variance for intention. Among the girls, three of the constructs (subjective norms, attitude, and barriers) entered the predictive equation in different ranking order for both intention and behavior and account for much of the variance.

The lack of association between intention and behavior might be explained by the concept of intention instability. Conner et al. found that intentions were stronger predictors of behavior when intentions were stable in adults eating a low-fat diet [35]. In the present study, youths' intention to eat healthy may be driven more by external cues and therefore constantly changing. As a result, behavior is affected to a greater extent by other factors and not intention. The TPB is based on the concept that the stronger the intention to perform a given behavior, the greater the likelihood that the person will perform that behavior [36]. In the current study, intention could not be considered strong because mean intention for boys was found to be 0.062 and girls was found to be 0.32 on a scale from -2 (low intention) to 2 (high intention). Forming strong intentions to eat healthy may not be a priority in youth and therefore do not affect eating behavior.

In comparing boys and girls, healthy eating behavior among boy was predicted by subjective norm and PBC, while among girls barriers, attitude, self-efficacy, and subjective norms predicted behavior. In addition, behavior in girls was positively associated with age and negatively associated with self-efficacy. It appears that as girls become older they are more likely to engage in healthy eating behavior, however a low self-efficacy also seems to be associated with healthy eating behavior. Results are puzzling and may be explained by girls' body dissatisfaction and distortions of a healthy body size. A study examining body perceptions among urban Native American youth found 61% of girls expressed a desire to be thinner compared to 41% of boys and 26% of girls selected the thinnest silhouette as the healthiest from range of eight varying sized silhouettes [7]. Compared to boys, girls had significantly higher mean values for intention and self-efficacy, and lower mean values for barriers to eat healthy. In another study examining food choice in youth, girls also reported greater intentions to eat fruit than boys [19]. Healthy eating promotion programs may benefit by offering gender separate activities that aim to enhance intention and self-efficacy in boys and reduce perceived barriers in girls.

Subjective norm and PBC explained 30% of the variance to eat healthy in boys while barriers, attitude, subjective norm, and self-efficacy explained 45% of the variance to eat healthy in girls. It appears that girls' behaviors are influenced by more factors and to a greater extent than boys. Subjective norm was the best predictor of healthy eating behavior in boys with family, television, after school programs, and friends being most instrumental in influencing eating behaviors. According to a review of the TPB's application to health related behaviors, the subjective norm construct often did not reach significance and had less influence on behavior than attitude and PBC constructs [37]. This was not found to be true in the sample of urban Native American boys in the present study. The primary social unit of Native Americans is the extended family and their culture is based on respect for elders and strong community ties [38]. A strong sense of family and community support may explain why youths' eating behaviors were directly affected by the subjective norm construct. For girls, similar to data from the total sample, barriers were most predictive of eating behavior with unavailability of healthy foods and taste cited as the greatest barriers. Including the entire Native American community in promotion strategies appears to be an appropriate method to enhance healthy eating behaviors in urban Native American youth, especially in boys and could also be effective in reducing perceived barriers regarding food availability in girls.

This study assessed self-reported eating behavior as part of the survey and as a result could have introduced inaccuracies. However, the authors feel that the this group of Native American youth, who are accustom to completing surveys during the after school program, were attentive when answering survey questions, thus minimizing potential problems. Additionally, youth were shown food models to increase the accuracy of their intake estimates. Measuring actual food intake is very labor intensive and dietary self-report is the method primarily used in studies assessing the TPB and eating practices in youth [15,16,18,19,39]. In addition, self-reported low-fat eating behaviors were better predicted by TPB than behavior measured by dietary observation in adults [27] and prediction was also found superior than observed behavior in a meta-analysis of the TPB [26]. Future studies may benefit from using multiple measurements of eating behavior to avoid potential inaccuracies from self-report.

This study only examined a sample of urban Native American youth and findings may not be true for all urban Native American youth. Tribe identity and regional location could alter the factors influencing healthy eating behavior in Native American youth. However, authors believe that findings are appropriate for basing future research and practice in populations of urban Native American youth.

## Conclusion

Findings indicate that TPB is useful for predicting factors directly related to healthy eating behavior but not for predicting the indirect effect of intention in a sample of urban Native American youth. This suggests that other factors besides intention are driving healthy eating behavior and can be used to develop intervention strategies to promote healthy eating practices in youth who overweight or at risk for overweight. Since barriers, attitude, subjective norm, and self-efficacy appear to be factors affecting healthy eating behaviors they should be incorporated in program design. Nutrition professionals should work with Native American community leaders and elders to provide sound nutritional knowledge to the entire community. This data also suggests that working through the family is important. Encouraging parents and caretakers to purchase and make healthy foods regularly available to their children could reduce youths' perceived barriers to healthy eating. Because the extended family and community are a valuable component of Native American culture, youth appear more willing to accept and follow dietary advice from members of their community. Gender differences may require the need for separate programs or at least special considerations for boys and girls. Boys who have lower self-efficacy but seem receptive to subjective norms, would benefit more from activities involving family and peers to increase healthy eating behaviors. Girls who are most affected by barriers to healthy eating, would benefit more from programs designed to increase the availability of healthy foods and promote the awareness that healthy foods are also tasty. Because of the alarming prevalence of obesity in urban Native American youth, future studies should continue to investigate the factors influencing obesity, such as eating and activity behavior, to identify the most effective way to solve this problem.

## Competing interests

The author(s) declare that they have no competing interests.
